# The Cytotoxicity and Genotoxicity of Bioactive Dental Materials

**DOI:** 10.3390/cells11203238

**Published:** 2022-10-15

**Authors:** Marta Kunert, Wioletta Rozpedek-Kaminska, Grzegorz Galita, Salvatore Sauro, Rim Bourgi, Louis Hardan, Ireneusz Majsterek, Monika Lukomska-Szymanska

**Affiliations:** 1 Department of General Dentistry, Medical University of Lodz, 92-213 Lodz, Poland; 2 Department of Clinical Chemistry and Biochemistry, Medical University of Lodz, 92-215 Lodz, Poland; 3 Dental Biomaterials and Minimally Invasive Dentistry, Departamento de Odontología, Facultad de Ciencias de la Salud, Universidad CEU-Cardenal Herrera C/Del Pozo ss/n, Alfara del Pa-triarca, 46115 Valencia, Spain; 4 Department of Therapeutic Dentistry, I. M. Sechenov First Moscow State Medical University, Moscow 119146, Russia; 5 Department of Restorative Dentistry, School of Dentistry, Saint-Joseph University, Beirut 1107 2180, Lebanon

**Keywords:** dental materials, vital pulp therapy, pulp capping, cytotoxicity, genotoxicity, flow cytometry

## Abstract

The promotion of biologically based treatment strategies in restorative dentistry is of paramount importance, as invasive treatments should be avoided to maintain the tooth’s vitality. This study aimed to assess the biocompatibility of commercially available bioactive materials that can be used for dental pulp capping. The study was performed with a monocyte/macrophage peripheral blood SC cell line (ATCC CRL-9855) on the following six specific bioactive materials: ProRoot MTA (Dentsply Sirona), MTA Angelus (Angelus), Biodentine (Septodont), TheraCal LC (Bisco), ACTIVA BioACTIVE (Pulpdent) and Predicta Bioactive Bulk (Parkell). The cytotoxicity of the investigated agents was measured using a resazurin-based cell viability assay, while the genotoxicity was evaluated using an alkaline comet assay. Additionally, flow cytometry (FC) apoptosis detection was conducted with a FITC (fluorescein isothiocyanate) Annexin V Apoptosis Detection Kit I. FC cell-cycle arrest assessment was carried out with propidium iodide staining. The results of this study showed no significant cytotoxicity and genotoxicity (*p* > 0.05) in ProRoot MTA, MTA Angelus, Biodentine, ACTIVA BioACTIVE and Predicta Bioactive. Conversely, TheraCal LC presented a significant decrease (*p* < 0.001). In conclusion, due to excellent biocompatibility and low cytotoxicity, MTA, Biodentine, ACTIVA BioACTIVE and Predicta Bioactive may be suitable for pulp capping treatments. On the other hand, due to the high cytotoxicity of TheraCal LC, its use should be avoided in vital pulp therapies.

## 1. Introduction

Minimally invasive biologically driven therapies that aim at preservation of the pulp vitality should be prioritized in contemporary dentistry. Dental materials used in vital pulp therapies (VPTs) have been extensively investigated in several recent studies, especially with regard to cytotoxicity, quality of reparative dentine bridge formation and protocols for greater bond strength to the tooth structure as well as to long-lasting final restorations [1,2,3,4,5]. In restorative dentistry, the paradigm has switched from total caries excavation techniques to selective caries excavation to avoid pulp exposure [6,7,8]. Indeed, direct and indirect pulp capping procedures are now the recommended clinical option to avoid root canal treatments. Dental pulp capping (PC) agents represent specific materials used as a protective layer and placed directly on the exposed pulp (direct pulp capping, DPC) or as a cavity liner placed over hypomineralized carious dentine (indirect pulp capping, IPC) in an attempt to induce its remineralization, and, therefore, protecting and preserving the pulp’s vitality. Ideally, such materials should be bioactive and stimulate the migration, proliferation and osteogenic differentiation of the cells and, at the same time, these must be highly biocompatible and not toxic to the pulp cells. Based on their composition, the PC agents investigated in this study can be assigned into the following four clinically significant groups: calcium silicate materials (CSMs), namely ProRoot MTA (Dentsply Sirona, York, PA, USA), MTA Angelus (Angelus, Londrina, Brazil), Biodentine (Septodont, Saint-Maur-des-Fossés, France); a light-cured calcium silicate-based material, TheraCal LC (Bisco, Schaumburg, IL, USA); a resin-modified glass ionomer (RMGIC) with improved resilience and physical properties, ACTIVA BioACTIVE (Pulpdent Corporation, Watertown, MA, USA) and a bioactive, dual cure, bulk fill resin composite, Predicta Bioactive Bulk (Parkell, Inc., Edgewood, NY, USA) [9,10].

ProRoot MTA has been available on the market since 1999, and it has been extensively studied and proven to be both bioactive and biocompatible [11,12]. To overcomedrawbacks, namely the long setting time as well as its high cost, the manufactures were urged to develop new types of MTA-based materials [13]. Offering the advantage of reduced final setting time —24–83 min [14,15] — significantly shorter than the original 228–261 min specific for ProRoot MTA [16,17], MTA Angelus was introduced. Addressing the difficult handling of MTA, novel PC agents have been designed in different consistencies for easier and more predictable clinical application. In 2011, Biodentine (BD) was released, and claimed to be a permanent, biocompatible dentine substitute. In contrast to traditional MTA, BD could be applied in one session in the whole volume of the cavity for an observation period or could be immediately followed by placement of the final restoration [18]. Its setting time, according to the manufacturer, is between 9 and 12 min; however, it was proven to set ultimately after 45 min [19]. On account of its advantages over MTA, such as reduced setting time and also solubility, mechanical properties and initial cohesiveness, BD has recently become a preferred agent for both DPC and IPC procedures although more long-term clinical studies are still needed to confirm Biodentine as the gold standard pulp capping agent [20].

In a search for a PC agent that could be used on one session and facilitate immediate and suitable bonding to restorative resins in final restorations, TheraCal LC was introduced [5,21]. Bioactivity in precipitating apatite-like crystals, cytotoxicity and biocompatibility have been indicated as the crucial features of pulp capping agents that directly affect the clinical outcome [8]. Overall, the current literature indicates that TheraCal LC is inferior to both MTA materials and BD, mainly because of inferior quality of calcific barrier formation, higher inflammatory effect, less favourable odontoblastic layer formation and lower calcium-releasing ability [22,23]. Those findings were due to the presence of resin monomer, which remained unpolymerized, so causing inflammation and toxicity to pulp tissue [24]. Moreover, heat generation during photopolymerization, which comes as a cost of preferable immediate setting, could still potentially induce unfavorable pulpal reactions when PC procedures are used [25]. Therefore, TheraCal should not be used for DPC [26].

Compared to both MTA and Biodentine, ACTIVA BioACTIVE as a resin-modified glass ionomer cement (RMGIC) has a favorable setting time, attributed to three setting mechanisms, with no delay in placing the final restoration. The use of traditional glass ionomer cement (GIC) is still limited due to its brittleness, sensitivity to water during initial setting and low compressive strength when compared to other restorative materials. In order to overcome these limitations, RMGIC was developed by modifying GIC with water-soluble resin. Therefore, in addition to the acid–base reaction, RMGICs offers benefits of photo-initiated polymerization, due to the presence of methacrylate monomers, photo-initiators and co-initiators. The main advantages of RMGICs are the ability for one visit treatment, adequate bond and also compressive strength to support the final restoration, preventing bacterial microleakage [27].The bioactive properties of ACTIVA BioACTIVE products are attributed to a mechanism by which the material responds to pH cycles and plays an active role in releasing and recharging of significant amounts of calcium, phosphate and fluoride from the GIC component and the modified calcium phosphate (MCP) contained within its composition. Nowadays, however, the release of biologically active ions from such materials and their precipitation in apatite-like crystals is more accurately termed ‘biointeractivity’ than bioactivity [22]. Nevertheless, the manufacturer recommends the use of ACTIVA BioACTIVE-BASE/LINER only in cases of no direct pulp exposure. Even though ACTIVA BioACTIVE supports human dental pulp stem cells’ (hDPSCs) proliferation, mineralization and attachment, further evaluation of its cytotoxicity needs to be conducted before being used for VPT [28]. Based on available research, it can be stated that, in cases of resin-containing materials, the risk of severe inflammation is higher than with CSMs; however, ACTIVA BioACTIVE-BASE/LINER showed some promising results in terms of more successful local and systemic tissue responses [29]. Literature does not provide sufficient evidence to substantiate its use in VPT; available data suggest that resin-free hydraulic calcium-silicate cements promote cell viability and bioactivity towards human cells better than resin-based agents, resulting in more successful clinical outcomes [30,31].

The latest development in the bioactive composites group, namely Predicta Bioactive Bulk, offers similar advantages to ACTIVA BioACTIVE. The release of calcium, phosphate and fluoride ions are responsible for stimulating mineral apatite formation and remineralization at the material–tooth interface. According to the safety data sheet Poly(2-hydroxyethyl methacrylate) (Poly-2-HEMA) monomers are used within the resin matrix with no potentially toxic BisGMA-based compounds [32]. However, very limited information is available regarding their characteristics, especially when exposed to pulp. Further research concerning their biocompatibility and possible cytotoxicity are required.

It is worth emphasizing that so far there have been no studies that evaluated the biocompatibility of the aforementioned materials using multiple in vitro assays, as presented in this article. In this study, a novel protocol was proposed for the comprehensive evaluation of toxicity by combining the following four different methodologies: cytotoxicity, genotoxicity, apoptosis detection and cell-cycle arrest assessment. All of the proposed methodologies were introduced to comprehensively evaluate immediate and postponed effects on human cells. As well as instant impairment of the cells studied by three independent tests, long-term genotoxicity may also impact clinical outcome, cell activity or cell structural changes. This influence was compared within the first 48 h post application for a holistic perspective on PC agents. Moreover, the present study is one of the first to evaluate novel bioactive materials, namely ACTIVA BioACTIVE and Predicta Bioactive Bulk, in terms of their toxicity towards human cells. Thus, this study aimed at assessing the biocompatibility of the few, most commonly used, bioactive materials, which may be used for vital pulp therapies. The objectives were accomplished with a monocyte/macrophage peripheral blood SC cell line (ATCC CRL-9855) at 24 h and 48 h. The null hypothesis was that the investigated bioactive materials would have the same toxicity towards SC cells.

## 2. Materials and Methods

### 2.1. Pulp Capping Materials

The following six bioactive materials were analyzed: ProRoot MTA (Dentsply Sirona, York, PA, USA), MTA Angelus (Angelus, Londrina, Brazil), Biodentine (Septodont, Saint-Maur-des-Fossés, France), TheraCal LC (Bisco, Schaumburg, IL, USA), ACTIVA BioACTIVE Liner (Pulpdent Corporation, Watertown, MA, USA) and Predicta Bioactive Bulk (Parkell, Inc., Edgewood, NY, USA).

### 2.2. Cell Line and Eluate Preparation

All of the in vitro analyses were performed in an experimental model using a commercially available monocyte/macrophage peripheral blood cell line—SC (ATCC CRL-9855) (ATCC; Manassas, VA, USA). Cell cultures were kept under standard conditions (37 °C; 5% pCO_2_; 95% humidity) according to the guidelines provided by the manufacturer. Cells were cultured in Iscove’s Modified Dulbecco’s Medium (IMDM) with 4-mML-glutamine adjusted to contain 1.5 g/L sodium bicarbonate (ATCC; Manassas, VA, USA) and supplemented with 0.05-mM 2-mercaptoethanol (Sigma-Aldrich Corp., St. Louis, MO, USA), 0.1-mM hypoxanthine and 0.016-mM thymidine (90%) (ATCC; Manassas, VA, USA), fetal bovine serum (10%) (ATCC; Manassas, VA, USA) and 1% penicillin/streptomycin solution (P/S) (ScienCell Research Laboratories, San Diego ad, CA, USA). Each cell culture was split when it reached 90–95% confluency. All of the tested cements were mixed according to manufacturer’s instructions and then cured in sterile hemi-sphere molds, r = 3.75 mm (surface area 1.33 cm^2^, volume of 140 μL). Immediately after reaching the setting time as provided by the manufacturer, the specimens were placed in Eppendorf tubes containing 1 mL of cell culture medium and were incubated for 24 and 48 h at 37 °C. The eluates were centrifugated for 5 min (2000 rpm) and then used for further analysis [33].

### 2.3. Cytotoxicity Analysis

The cytotoxicity of the materials used in VPT was measured using a commercially available colorimetric, resazurin (7-Hydroxy3H-phenoxazin-3-one 10-oxide)-based assay kit (Sigma Aldrich Corp., St. Louis, MO, USA), which detects cellular metabolic activity. Resazurin is irreversibly reduced to a pink color and bright red fluorescent resorufin by dehydrogenase enzymes only in metabolically active cells. The whole experiments were performed in triplicate with similar results. SC cells were seeded in 96 well plates by adding 50 μL (8 × 10^3^/well) cultured cell suspension and 50 μL of the tested specimens’ eluate into the complete IMDM medium. Untreated cells cultured in a complete IMDM medium were used as a negative control, whereas cells incubated with 100% dimethyl sulfoxide (DMSO) comprised a positive control. Cells were incubated for 24 h and 48 h at 37 °C, respectively. After the incubation time, the plates with cells were centrifuged and the supernatants were removed. Subsequently, 100 μL of the 10% solution of resazurin in complete IMDM medium was added to each well. After 4 h incubation at 37 °C absorbance was measured at a wavelength of 600 nm and at a reference wavelength of 690 nm using a Synergy HT (high-throughput) spectrophotometer (BioTek, Vermont, VT, USA) [34].

### 2.4. Genotoxicity Assessment

The genotoxicity of the tested materials was evaluated by an alkaline version of the comet assay, in order to analyze deoxyribonucleic acid (DNA) damage in specific cells. Assays were prepared in 12 well plates by adding 500 μL (5 × 10^4^ cells/well) of complete medium and 500 μL of previously prepared eluates. Cells suspended in highly toxic 10% DMSO (Sigma-Aldrich Corp., St. Louis, MO, USA) constituted a positive control, whereas cells suspended in 1 mL of complete culture medium constituted a negative control. Subsequently, specimens were incubated for 24 h and 48 h. Cells suspended in 0.37% low melting point (LMP) agarose (Sigma-Aldrich Corp., St. Louis, MO, USA) were placed on microscope slides that were previously coated with normal melting point (NMP) agarose (Sigma-Aldrich Corp., St. Louis, MO, USA). Preparations were incubated in lysis buffer at pH 10 (2.5-M NaCl, 10-mM Tris, 100-mM EDTA), containing TritonX-100 (Sigma-Aldrich Corp., St. Louis, MO, USA), at a final concentration of 1% at 4 °C for 60 min. After 1 h incubation, the preparations were incubated in development buffer (300-mM NaOH, 1-mM EDTA) for 20 min at 4 °C and this was followed by electrophoresis (32 mA, 17 V, 20 min) at 4 °C in electrophoretic buffer (30-mMNaOH, 1-mM EDTA). Eventually, the preparations were stained with a 4′,6-diamidino-2-phenylindole (DAPI) fluorescent dye and the obtained data were analyzed with a fluorescent microscope. Evaluation of genotoxicity of the tested materials was conducted based on the percentage of DNA in the comet tail [35].

### 2.5. Apoptosis Detection

Apoptotic cell death induced by the eluates of the tested compounds was assessed using a fluorescein isothiocyanate FITC Annexin V Apoptosis Detection Kit I (FITC Annexin V Apoptosis Detection Kit I, BD Bioscences, NJ, USA). Assays were prepared in 12 well plates by adding 500 μL (1 × 10^6^ cells/well) of complete medium and 500 μL of prepared eluates, and incubated for 24 h and 48 h. Cells treated with staurosporine (Sigma-Aldrich Corp., St. Louis, MO, USA) at a concentration of 1 μM for 16 h constituted a positive control. The negative control comprised cells suspended in the complete culture medium and incubated for 24 h and 48 h. Subsequently, cells were washed twice with cold phosphate buffered saline (PBS) (Sigma-Aldrich Corp., St. Louis, MO, USA) and then double stained with annexin V as a marker of early apoptosis, and propidium iodide (PI) as a marker of cell membrane disintegration, necrosis and late apoptosis. The percentage of apoptotic cells was calculated by flow cytometry (FC) using a CytoFLEX (Beckman Coulter, Brea, CA, USA). Obtained data were analyzed using Kaluza analysis 1.5 A software (Beckman Coulter) [33].

### 2.6. Cell Cycle Analysis

The analysis of the cell cycle was performed by FC using PI staining. Assays were prepared in 12 well plates by adding 500 μL (1 × 10^6^ cells/well) of complete medium and 500 μL of prepared eluates and then incubated for 24 and 48 h. Cells treated with 1 μM of the cell cycle arresting factor nocodazole (Sigma-Aldrich Corp., St. Louis, MO, USA) for 16 h constituted a positive control, whereas cells cultured in a complete medium for 24 h and 48 h constituted a negative control. Cells were washed twice with cold PBS (Sigma-Aldrich Corp., St. Louis, MO, USA) and then fixed with ice-cold 70% ethanol at −20 °C for 20 min. Afterwards, cells were treated with RNase A DNase & Protease-free (10 mg/mL) (Canvax Biotech, Córdoba, Spain) and incubated at 37 °C for 1 h before staining with PI solution (10 μg/mL) (Sigma-Aldrich Corp., St. Louis, MO, USA). After a 30 min incubation at 4 °C, the percentage of cells in each cell cycle phase were assessed using Kaluza analysis 1.5 A software (Beckman Coulter). On the DNA content histograms, the number of cells was plotted on the y-axis, whereas the DNA content, as measured by PI fluorescence, was depicted on the x-axis [35].

### 2.7. Statistical Analysis

Statistical analysis was performed using Statistica 13 (StatSoft, Krakow, Poland). A Shapiro–Wilk test was performed to test normality. All statistical data, except for the comet assay test, were normally distributed, therefore statistical analysis between the two groups was performed using Student’s t-test. No normal distribution was obtained in the comet assay analysis, thus statistical analysis of the two groups was conducted with a Mann–Whitney rank sum test. All analyses in each experiment were based upon the results of three independent tests. Statistically significant differences are shown on graphs as follows: * *p* < 0.05; ** *p* < 0.01; *** *p* < 0.001 vs. negative controls.

## 3. Results

### 3.1. Analysis of the Cytotoxicity of the Pulp Capping Agents

**Figure 1:**
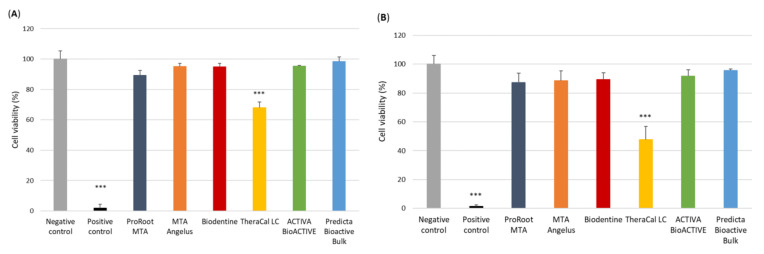
Cytotoxicity of the investigated pulp capping materials. Tests performed using resazurin-based cell viability assay after 24 h (**A**) and 48 h incubation (**B**) of cells with the tested compounds. The positive control was represented by the cells incubated with 100% DMSO, while the negative control cells were cultured in a complete IMDM medium. Statistical significance on the graphs: *** *p* < 0.001 versus negative control.

The cytotoxicity analysis performed using the resazurin-based assay kit showed significant differences in the cytotoxic properties of one of the investigated compound eluates. The results obtained showed that, both after 24 h and 48 h of incubation with monocyte/macrophage peripheral blood cell line (SC) cells, TheraCal LC was the only material to induce a significant decrease in cell viability compared to the control groups (Figure 1A,B) (*p* < 0.001).

### 3.2. Analysis of the Genotoxicity of the Pulp Capping Agents

**Figure 2:**
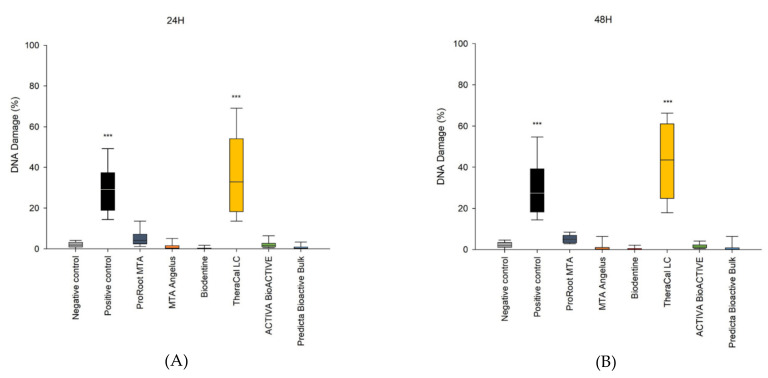
Genotoxicity of the investigated materials. Analysis was performed using an alkaline version of the comet assay after 24 h (**A**) and 48 h (**B**) incubation of cells with the tested compounds. Cells suspended in 10% DMSO were used for the positive control. Cells suspended in 1 mL of complete culture medium were employed as the negative control. Statistical significance on the graphs: *** *p* < 0.001 versus negative control.

A significant increase in DNA damage was observed after both 24 h and 48 h incubation in the SC cells treated with TheraCal LC (Figure 2A,B) (*p* < 0.001). All of the other tested materials induced no significant DNA damage (Figure 2A,B) (*p* > 0.05).

### 3.3. Apoptosis Detection by FITC Annexin V/PI Double Staining of the Pulp Capping Agents

**Figure 3:**
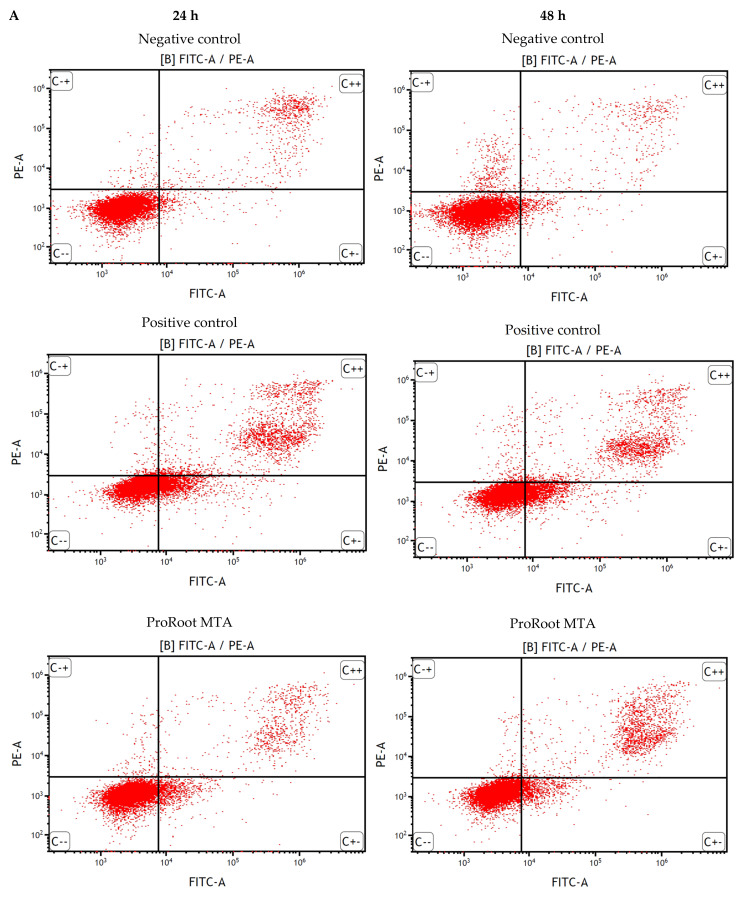

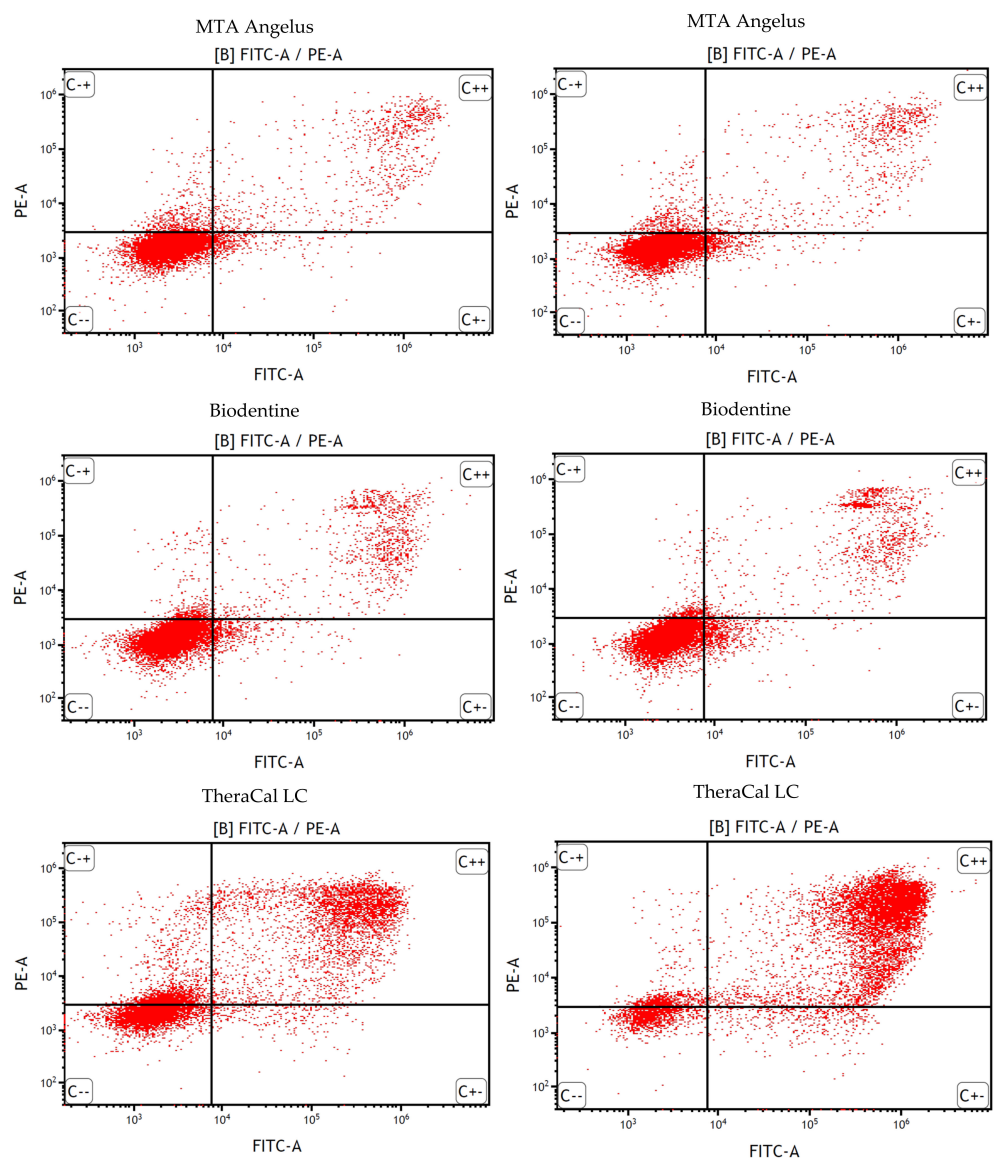

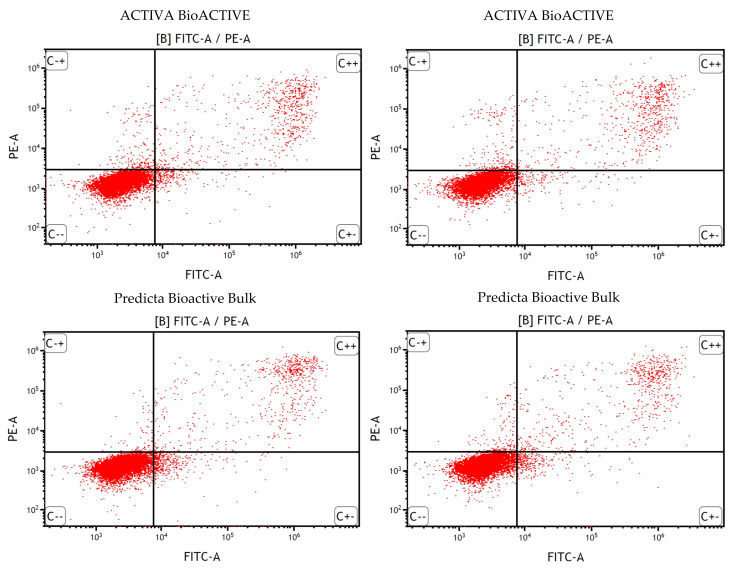

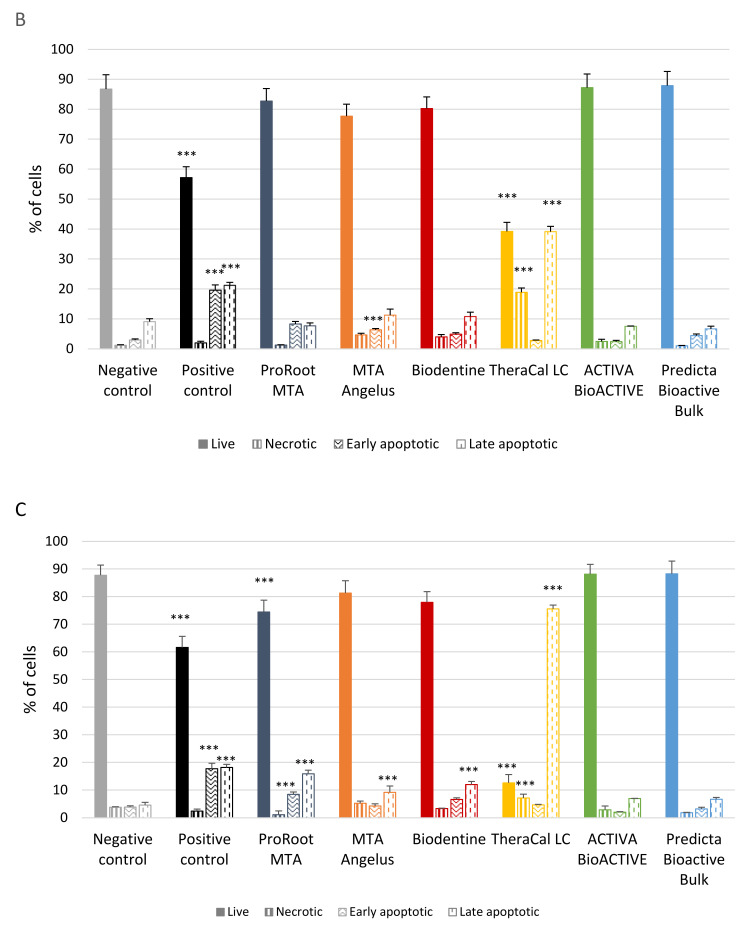
Flow cytometric FITC annexin V/propidium iodide (PI) double staining analysis of apoptosis after 24 h and 48 h incubation of cells with the tested compounds. Dot plot graphs indicate share of viable (FITC annexin V negative, PI negative), early apoptotic (FITC annexin V positive, PI negative) late apoptotic (FITC annexin V positive, PI positive) and necrotic (FITC annexin V negative, PI positive) cells (**A**). Percentage of cells in each group after 24 h are presented below (**B**) and 48 h incubation (**C**). ***—statistically significant difference (*p* < 0.001).

After 24 h of incubation, TheraCal LC significantly induced apoptosis, with even poorer results after 48 h of incubation (approximately 42% and 80% of cells were at the early and late stages of apoptosis, respectively). Other PC agents induced no significant apoptosis, although ProRoot MTA, after 48 h of incubation, showed some toxicity (74.49% of the cells vital). Additionally, none of the tested compounds evoked a significant increase in the level of necrotic SC cells (Figure 3) (*p* > 0.05).

### 3.4. Analysis of the Cell Cycle Progression by PI Staining of the Pulp Capping Agents

**Figure 4:**
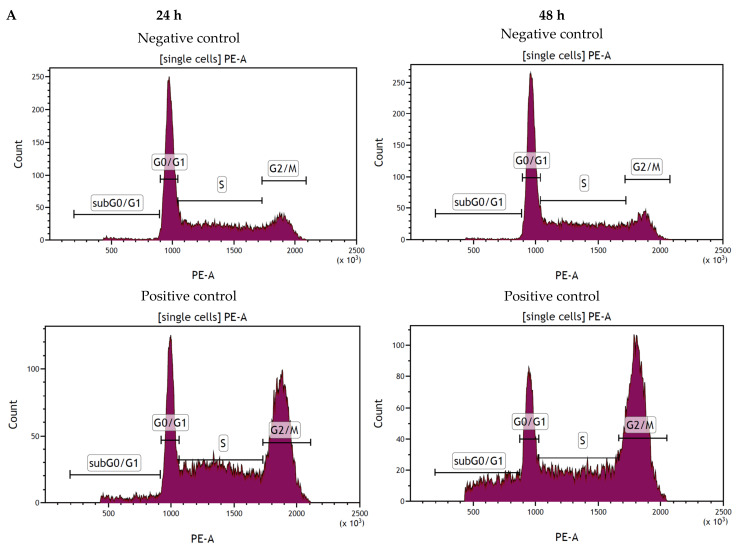

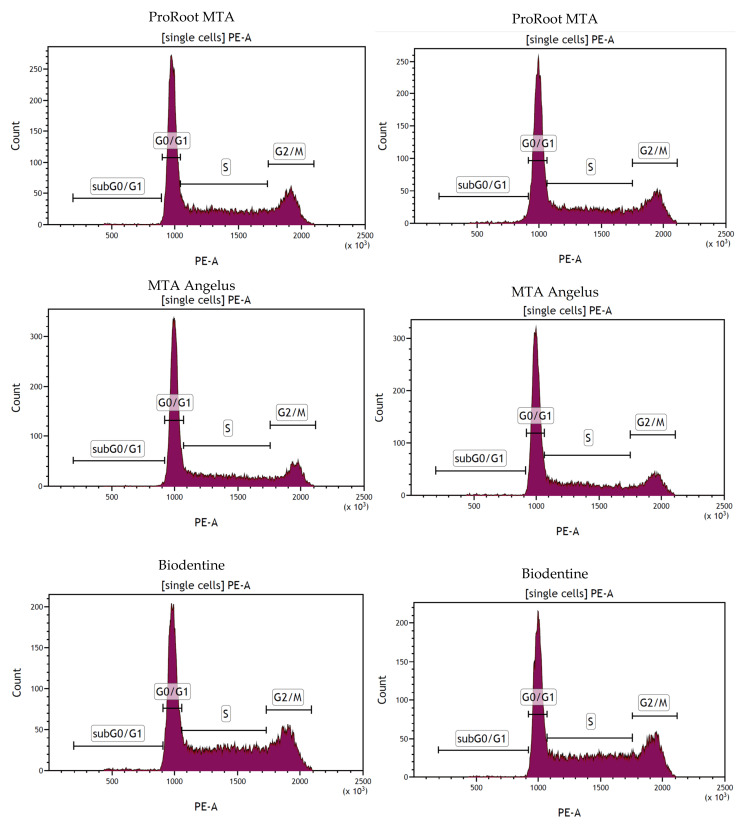

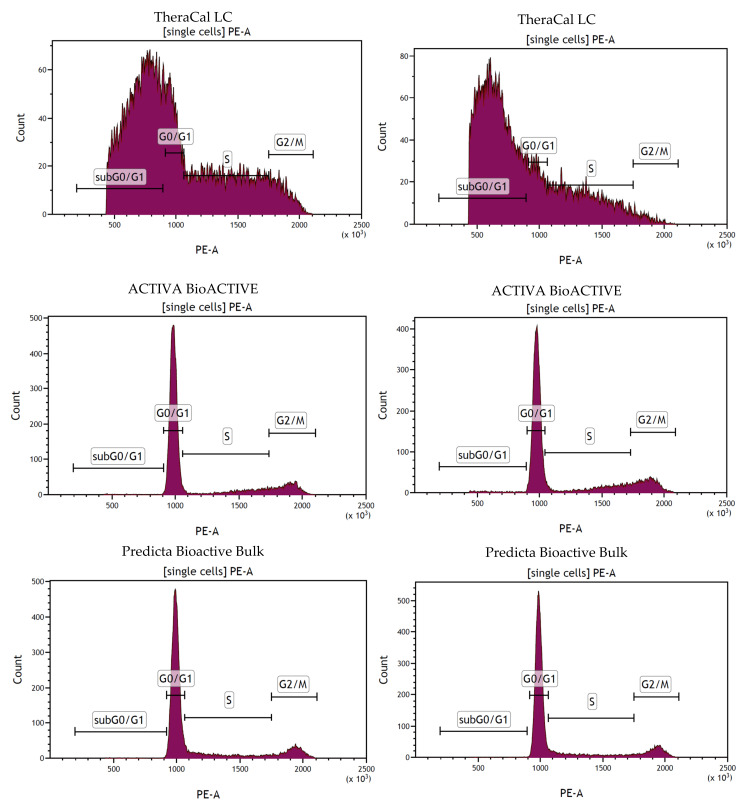

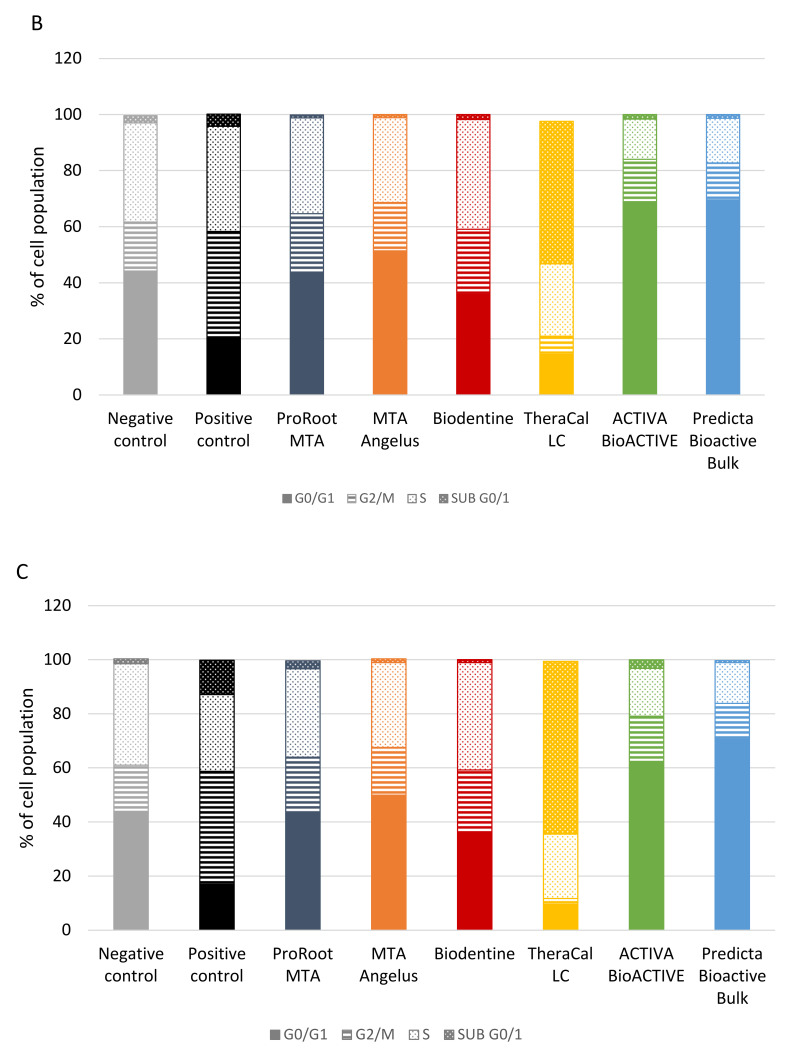
Flow cytometry (FC) analysis of cell cycle progression using propidium iodide (PI) staining after 24 and 48 h incubation (**A**) of cells with the tested compounds. Percentage of cells in each group after 24 h are presented below (**B**) and 48 h incubation (**C**). Cells treated with 1 µM nocodazole constituted a positive control. Cells cultured in the complete medium represented the negative control.

The cell cycle progression of the SC cells treated with ProRoot MTA, MTA Angelus, Biodentine, ACTIVA BioACTIVE and Predicta Bioactive Bulk was similar to the SC cells cultured in the complete medium (*p* > 0.05). The TheraCal LC, after 24 h and 48 h incubation, triggered a significant rise in the percentage of SC cells in the sub-G0/G1 phase and a significant reduction in the percentage of cells in the G1 phase of the cell cycle, as compared to the negative control (Figure 4).

## 4. Discussion

In the present study, the tested materials showed significantly different toxicity. While ProRoot MTA, MTA Angelus, Biodentine, ACTIVA BioACTIVE and Predicta Bioactive showed no significant cytotoxicity and genotoxicity, according to the controls used, TheraCal LC significantly decreased cell viability and presented significant DNA damage in the comet assay. Hence, the null hypothesis tested in this in vitro study was rejected. The results of the current study are in accordance with other investigations based on different methods such as MTT reduction assay [3,36,37]. Similar results were also obtained in the apoptosis detection test performed using FC. Biodentine, ACTIVA BioACTIVE and Predicta Bioactive showed no significant increases in apoptosis in the tested cell line, although ProRoot MTA and MTA Angelus presented some toxicity, while TheraCal LC significantly boosted the percentage of cells in the early and late stages of apoptosis. The cell cycle analysis with FC showed a significant escalation in the sub-G0/G1 phase in cells that were treated with the TheraCal LC eluate, indicating that there was a higher number of dead cells compared to both controls and the other tested materials. All of the assays performed in this study confirmed the hypothesis of high cytotoxicity of the resin-based TheraCal LC in VPT. Moreover, the present results are in accordance with some previous studies also performed on hDPs and hDPSCs [36,37,38]. Therefore, the use of such materials should be limited only to indirect pulp capping [26].

Differences in toxicity could be related to the presence of monomers and also to the ratio of the compounds in specific pulp capping agents. It is therefore important to emphasise that the biocompatibility of pulp protection materials is the most important factor when these have direct or indirect contact with the pulp. According to the safety data sheet, the resin matrix of Theracal LC contains polyethylene glycol dimethacrylate and BisGMA, which, especially unpolymerized, may impact its toxicity [39]. Due to its low shrinkage, sufficient mechanical and esthetic properties but also excellent adhesion to enamel [40], Bis-GMA is the base monomer of the majority of resins used in dentistry [41]. Unfortunately, this monomer is characterized by a toxic effect on endocrine cells, even in low concentrations; therefore the release of bisphenol A (BPA) is of great interest in recent research [42]. Indeed, BPA mimics the behavior of the natural hormone estradiol by interacting with estrogen receptors [43]. Furthermore, it is known that BisGMA, among the monomers used in dental materials, has relatively high cytotoxicity as its hydrolysis products may induce the loss of cell membrane permeability and it also exhibits proinflammatory, carcinogenic, and even mutagenic effects [44,45]. However, pure BPA is not used as a monomer in dentistry, but, instead, occurs as impurity of the synthesis process of derivates like Bis-GMA, thus only traces of such a monomer can leach from resin composites to the tissues. The conversion rate of the monomers into BPA ranges between 0.0003% and 0.0025% and there is an increase when the resins are exposed to saliva and to the degrading action of *S. mutans* [41]. Removal of the oxygen inhibition layer and prolonged light-curing procedures can reduce the risk of elution of unpolymerized monomers and relative cytotoxicity [43,46]. Nevertheless, excessive photopolymerization could potentially induce adverse pulpal effects when used in pulp capping procedures, due to heat generation [25]. This latter effect is supposed to be less evident in the case of ACTIVA BioACTIVE, as well as Predicta Bioactive, since they are dual curing materials. Moreover, self-curing materials applied in deep cavities may have fewer issues related to a lack of polymerization, with a lower presence of potentially toxic unpolymerized monomers. Moreover, the absence of Bis-GMA within the composition of ACTIVA BioACTIVE and Predicta Bioactive could also be a reason for the low cytotoxicity and genotoxicity observed in the current study. However, novel bioactive materials require further investigation, since their exact compositions are not yet clear, therefore it is quite difficult to understand their real bioactive properties and the possible synergistic effects these could have on the biocompatibility and the cytotoxicity.

The limitations of the present investigation are related to the in vitro character of the experiments performed to accomplish the aims of this study. The model shows limited information on the number of residual monomers and other components in the mixture of the pulp capping agent that can infiltrate to the pulp tissue. There are several variables that could affect this leaching, such as the thickness of dentine or the exposed surface area. The cytotoxicity data may also vary depending on the volume ratio of material versus the extract medium [3,30]. In this study, the equivalent of 140 μL of all of the materials was used and 500 μL eluates were prepared, then mixed with complete medium at ratio of 1:1. In the clinical practice, the tested materials are used in different amounts because of their flexural strength. In cases of DPC, ProRoot MTA, MTA Angelus and TheraCal LC would be placed locally on the pulp exposure with a maximum of 1 mm margin diameter, while Biodentine, ACTIVA BioACTIVE and Predicta Bioactive could be placed as a liner or even in the full cavity volume; this difference could also affect the impact on the pulp cells.

There are different cell types that may be used in in vitro studies to assess the cytotoxicity and genotoxicity of dental materials. Some studies of the toxicity of materials used in VPT were conducted on cells derived from pulp, namely human dental pulp cells (hDPs) and human dental pulp stem cells (hDPSCs) [12,36,37,38]. However, cells cultured in vitro over several generations may undergo genomic transformations and/or mutations, therefore they might be unreliable for studies of DNA damage. The most favorable studies of genotoxicity are those using diploid cell lines such as human leukocytes [47]. The SC cell line was chosen as the preferable in both the cytotoxicity and genotoxicity studies that were performed in this study. The choice of cell line was also in accordance with PN-EN ISO 10993-11:2018, since systemic toxicity should be also evaluated on monocyte/macrophage cells as a stage in pre-clinical research for biological materials such as the evaluated pulp capping materials [48]. The reliability of the methods used in this study is high, as the corresponding results in different tests based on different molecular mechanisms of toxicity, cytotoxicity, genotoxicity, apoptosis induction and cell cycle arrest were in line with available research performed on hDPs and hDPSCs.

The in vitro study has a specific limitation as it cannot replicate the clinical performance of the materials, as they can be present in the cavity for number of years. Thus, the in vitro model does not take into consideration the long-term effects, the absence of a dentine barrier, the immune response present in human tissues or factors like age of the patient, which have been proven to be significant in terms of the success rate of VPT [49]. However, the in vitro biological properties of the tested materials described in the present study may act as a preliminary assessment of their potential biological behavior. The comparison of the in vitro tests, which allow the evaluation of many samples simultaneously, and clinical performance of the materials used in pulp capping procedures is crucial and must be investigated in further studies.

This study demonstrates the favorable in vitro biocompatibility and bioactive properties of ProRoot MTA, MTA Angelus, Biodentine, ACTIVA BioACTIVE and Predicta Bioactive, suggesting their superior regenerative potential compared with TheraCal LC. Novel bioactive materials, namely ACTIVA BioACTIVE and Predicta Bioactive may be a promising alternative to clinically proven bioceramics, although to date there is not sufficient evidence to corroborate its use in vital pulp therapies when placed directly in contact with the pulp tissue, especially as manufacturers recommend applying pulpal protection to deep excavation areas. Bioactivity, lack of cytotoxicity and genotoxicity and absence of potentially toxic monomers in their composition are favourable characteristics in cases of indirect pulp capping procedures, but their performance in DPC is still unclear and it is necessary to perform further investigation.

It is worth emphasizing that, to date, no studies have evaluated the biocompatibility of the materials used in VPT using multiple in vitro assays, as presented in this research. Moreover, new bioactive materials have still not yet been studied using the aforementioned techniques. A comparison of the recently developed ACTIVA BioACTIVE and Predicta Bioactive to the more widely examined CSMs has given a preliminary perspective on the role that those materials could have in pulp capping procedures. Furthermore, the application of comprehensive techniques such as the resazurin assay, comet assay and analysis of the level of apoptosis and cell cycle distribution via the FC may lead to the establishment of novel testing protocols in dental materials science.

## 5. Conclusions

It can be concluded that PC agents vary in both their cytotoxic and genotoxic effects on human monocyte/macrophage peripheral blood SC cells. While ProRoot MTA, MTA Angelus, Biodentine, ACTIVA BioACTIVE and Predicta Bioactive showed no significant cytotoxicity and genotoxicity compared to the controls used, TheraCal LC significantly decreased the cell viability and presented significant DNA damage in the comet assay. Similar results were obtained in the apoptosis detection test performed using FC. Biodentine, ACTIVA BioACTIVE and Predicta Bioactive showed no significant increase in apoptosis in the tested cell line, although ProRoot MTA and MTA Angelus showed minimal toxicity, while TheraCal LC significantly increased the percentage of cells in the early and late stages of apoptosis. The cell cycle analysis with FC showed a significant increase in the sub-G0/G1 phase in cells that were treated with the TheraCal LC eluate.

## Data Availability

The data presented in this study are available on request from the corresponding author.
